# Etymologia: Prion

**DOI:** 10.3201/eid1801.ET1801

**Published:** 2012-01

**Authors:** Nancy Männikkö

**Keywords:** etymologia, prion, protein, infection, Prusiner, history

## Prion [pri′on, pre′on]

From *pro*tein + *in*fection. Nobel laureate Stanley B. Prusiner, American neurologist and biochemist, coined the word prion in 1982 to describe the noninfectious agents he proposed as the cause of scrapie. Prusiner noted that, “Because the dominant characteristics of the scrapie agent resemble those of a protein, an acronym is introduced to emphasize this feature… the term ‘prion’ (pronounced *pree-on*) is suggested.” Prions are now recognized as etiologic agents of other transmissible spongiform encepalopathies, including bovine spongiform encephalopathy and Creutzfeld-Jakob disease.Sources: Dorland’s Illustrated Medical Dictionary. 31st ed. Philadelphia: Saunders; 2007; The Nobel Prize in Physiology or Medicine 1997: Stanley B. Prusiner [cited 2011 Oct 25]; http://nobelprize.org/nobel_prizes/medicine/laureates/1997/prusiner-autobio.html; Prusiner SB. Novel proteinaceous infectious particles cause scrapie. Science. 1982;216:136–44

